# Living with chronic kidney disease: future perspectives and prognostic needs of patients - a qualitative study

**DOI:** 10.1186/s12882-025-04243-8

**Published:** 2025-07-01

**Authors:** Jet Milders, Yvette Meuleman, Chava L. Ramspek, Willem Jan W. Bos, Wieneke M. Michels, Ype de Jong, Friedo W. Dekker, Merel van Diepen

**Affiliations:** 1 Department of Clinical Epidemiology, Leiden University Medical Center, Leiden, The Netherlands; 2 Parnassia Groep, The Hague, The Netherlands; 3 Department of Internal Medicine, Leiden University Medical Center, Leiden, The Netherlands; 4 Santeon, Utrecht, The Netherlands; 5 Department of Internal Medicine, St. Antonius Hospital, Nieuwegein, The Netherlands; 6 Department of Thrombosis and Haemostasis, Leiden University Medical Center, Leiden, The Netherlands

## Abstract

**Background:**

Individualized prognostic information can help patients with chronic kidney disease (CKD) understand and prepare for their future, facilitating informed shared decision-making. While research has indicated that CKD patients want more information about their future, little is known about their specific prognostic needs. Therefore, this study aims to explore how patients with CKD perceive their future and what their prognostic needs are.

**Methods:**

A survey was constructed with patient representatives, and distributed amongst adult CKD patients (all stages) through patient associations and healthcare professionals in two Dutch hospitals. Following an exploratory-descriptive qualitative approach, answers to four open-ended questions of 163 patients were analysed inductively using thematic analysis.

**Results:**

Patients described a wide range of emotions when thinking about their future with CKD, including negative emotions like uncertainty, fear, sadness, and to a lesser extent, anger. However, some patients maintained emotional neutrality or described experiencing positive emotions like calmness, hope and trust. Additionally, patients had diverse prognostic needs, focusing on different topics like CKD treatment, kidney disease progression, self-management, symptoms, life expectancy and life participation. While most patients wanted more personalized prognostic information on these topics, some felt like they were already sufficiently informed about their future or preferred to live in the present moment.

**Conclusions:**

Our findings show that CKD patients experience a wide variety of emotions regarding their future. Despite conversations about their future taking place in nephrological practice, there remain unmet questions regarding the future. These results underline the importance of adopting a personalized approach when discussing the future with CKD patients, acknowledging and taking the diverse emotional responses and individual preferences into account.

**Supplementary Information:**

The online version contains supplementary material available at 10.1186/s12882-025-04243-8.

## Introduction

Living with chronic kidney disease (CKD) comes with a high disease burden, as patients can experience many symptoms like pain, pruritus, fatigue, mental and sexual problems [1–8]. These symptoms can seriously disrupt patients’ daily (social) activities and diminish their quality of life. In advanced CKD stages, treatment burden increases as patients often require intensive treatments, and are confronted with a heightened risk of adverse outcomes like hospitalization and death [9–12]. Faced with these challenges, CKD patients often experience uncertainty regarding their prognosis, and feelings of hopelessness and fear [13]. 

The CKD course varies for every patient, making it difficult to foresee the disease impact on patients’ lives in the future. Prognostic information tailored to the individual patient could provide insight into *what* to expect, and *when* these outcomes may occur, providing reassurance and time to prepare for the future [14, 15]. Additionally, well-informed patients are essential for shared-decision making, for example, when considering different treatment options and the impact each option could have on their future.

Literature shows that patients desire more prognostic information earlier on in their disease trajectory. For example, CKD patients report wishing that they were better educated about self-management, prognosis, medication and kidney function to help them regain a sense of control over their disease. Additionally, they express interest in receiving personalized risks of reaching certain outcomes, and timeframes within which outcomes may occur [13, 14, 16]. Similar needs have been identified in other chronic illnesses such as heart failure, cancer, and chronic obstructive pulmonary disease (COPD), where patients also express a wish for clearer, more personalized prognostic information [17–19]. However, knowledge about the specifics of their prognostic needs is lacking: which information do they want, how do they want to receive it, and when do they desire it most? Our previous quantitative study confirmed that patients with CKD desire more prognostic information about their future and about which topics (e.g. kidney function, energy levels and quality of life) [20]. Building on these findings, we now seek to obtain an in-depth understanding of the reasons and stories behind these prognostic needs, the broader context in which they arise, and patients’ emotions regarding their future. To achieve this, we qualitatively analysed the answers to the open-ended survey questions, aiming to explore two research questions: (1) How do patients with CKD view their future? and (2) What are the prognostic needs of patients regarding their future with CKD?

## Materials and methods

This qualitative study uses an exploratory-descriptive approach, aiming to gain a deeper understanding of patients’ experiences, perspectives, and meanings without predefined hypotheses [21, 22]. To ensure transparent reporting, we followed the Checklist for Reporting of Survey Studies (CROSS; Table S1 [23]) and where applicable, the Consolidated criteria for reporting qualitative studies (COREQ) [24]. 

### Ethics

This study was not subject to the Medical Research Involving Human Subjects Acts (WMO) as declared by the scientific committee of the department of Clinical Epidemiology at Leiden University Medical Center (LUMC) in The Netherlands [25]. 

### Survey development and testing

A survey was constructed by an expert panel of researchers (JM, CLR, FWD, MvD) and nephrologists (WJWB, WMM) with experience in surveys, patient-reported outcomes, qualitative and prognostic research; and by patient representatives from the Dutch Kidney Patients Association (NVN) to ensure comprehensibility of the survey [26]. Castor Electronic Data Capture System was used for the online survey. A paper version was constructed to allow patients without internet access to participate. Responses from the paper surveys were manually entered into Castor. During a two-phase pilot, the survey was tested in collaboration with NVN-volunteers (Supplementary Materials). The final survey contained questions on 1) demographics, 2) considerations about the future, and 3) prognostic needs. See Supplementary Materials for the full survey (translated from Dutch to English) and accompanying informational letter. Answers to four open-ended questions were analysed: A) What emotions do you experience when you think about your future with CKD?; B) Have you ever discussed your future with CKD with your treating physician? If yes, could you elaborate on that conversation?; C) What would you like to know about your future with CKD?; and D) If you think back to a year or two ago, what would you have wanted to know about your life with CKD that you know now?. Answers to question A were used to answer RQ1 “How do patients with CKD view their future?”; questions C and D were used to answer RQ2 “What are the prognostic needs of patients regarding their future with CKD?“, and question B was used to provide contextual information about current prognostic information provision.

### Study population and recruitment

All adult patients with CKD could participate, including those receiving KRT (dialysis or transplantation). We used convenience and purposive sampling, first deploying the survey through regional and national kidney patients associations (Diavaria and NVN). Since these sources mainly reached transplanted patients, the survey was also distributed at two Dutch hospitals (LUMC and Sint Antonius Hospital Nieuwegein) via nephrologists, nurse practitioners and one researcher (JM) to reach a more diverse and representative sample of the Dutch CKD population.

### Data collection and analysis

Patients completed the survey between October 17th 2022 and March 13th 2023. Surveys were gathered anonymously and duplicates (i.e. patients that started the survey multiple times) were removed. Questions were analysed using available case analysis, as completing open-ended questions was optional. Answers were analysed inductively following all six phases of thematic analysis [24–26]. In phase 1 (familiarization with data), one researcher (JM) read all responses multiple times and made initial notes. In phase 2 (inductive coding), meaningful segments of text were systematically coded and organized in Atlas.ti (v8.3.0-2024-07-29) by JM, with early discussions about codes and interpretations taking place in consultation with two other researchers (YM, MvD). In phase 3 (searching for themes), codes were clustered into initial themes. In phase 4 (reviewing themes), the thematic structure was refined through discussions with two other researchers (YM, MvD), ensuring that the themes captured key patterns in the data in relation to the research questions. In phase 5 (defining and naming themes), themes were clearly defined and named, and in phase 6 (writing up), findings were described using illustrative quotes. Illustrative quotes were translated from Dutch to English using back translation. Analyses were performed using Atlas.ti (Atlas, Berlin, v8.3.0-2024-07-29).

## Results

### Response and participant characteristics

Through the patient organizations, 530 patients were contacted of which 108 (20.4%) completed the survey fully and 10 individuals (1.9%) partially. A total of 16 patients from LUMC completed the survey, and 28 complete and 1 partial survey(s) were filled in at the Sint Antonius Hospital. In total, six duplicates were removed. Open-ended question A, B, C and D (see Methods) were filled in by 137 (84.1%), 58 (35.6%), 131 (80.4%) and 125 (76.7%) patients, respectively.

In Table 1, participant characteristics are presented. Approximately half of the patients were female (50.9%), and the mean age was 63.9 years (SD 12.0). The majority of patients had undergone a kidney transplantation (56.4%), 16.0% was receiving dialysis, and 27.6% had CKD without receiving KRT.

**Table 1:** General characteristics of patients

	Total (*n* = 163)
Patient recruitment source, n (%)	
National Dutch Kidney Patients Association (NVN)	100 (61.3%)
Local Kidney Patients Association Hollands-midden (Diavaria)	18 (11.0%)
Leiden University Medical Center	16 (9.8%)
Sint Antonius Hospital Nieuwegein	29 (17.8%)
Age (mean, SD)	63.9 (12.0)
Gender (female, n (%))	83 (50.9%)
CKD patients without KRT	45 (27.6%)
Dialysis modality/type, n (%)	26 (16.0%)
Haemodialysis in hospital	24 (92.3%)
Haemodialysis at home	1 (3.8%)
Peritoneal dialysis	1 (3.8%)
Kidney transplantation, n (%)	92 (56.4%)
Education level, n (%)*	
Low	41 (25.2%)
Medium	36 (22.1%)
High	84 (51.5%)
Other	1 (0.6%)
Living situation, n (%)	
Alone	35 (21.5%)
Together with a partner	114 (69.9%)
Child(ren) living at home	27 (16.6%)
Care facility	3 (1.8%)
Other**	4 (2.5%)
Cause of kidney disease, n (%)	
Diabetes mellitus	11 (6.7%)
Vascular disease	15 (9.2%)
Glomerulonephritis	16 (9.8%)
Pyelonephritis, kidney damage by medication or nephrolithiasis	9 (5.5%)
Polycystic kidney disease	35 (21.5%)
Autoimmune disease	16 (9.8%)
Cancer	3 (1.8%)
Unknown	31 (19.0%)
Other	27 (16.6%)
Self-reported kidney function (eGFR), n (%)	
> 60 ml/min/1.73 m^2^	30 (18.4%)
45–59 ml/min/1.73 m^2^	33 (20.2%)
30–44 ml/min/1.73 m^2^	32 (19.6%)
15–29 ml/min/1.73 m^2^	25 (15.3%)
< 15 ml/min/1.73 m^2^	30 (18.4%)
Unknown	13 (8.0%)
Time since CKD diagnosis, n (%)	
0–4 years	17 (10.4%)
5-10 years	25 (15.3%)
> 10 years	121 (74.2%)

Some questions allowed multiple answers, meaning that percentages may add up to more than 100%

*Education levels were categorized based on the International Standard Classification of Education (ISCED) [50], **Patients that chose “Other” lived with parent(s), a housemate or spent the weekends with a long-distance partner

## How do patients with Ckd view their future? (RQ1)

**Fig. 1:**
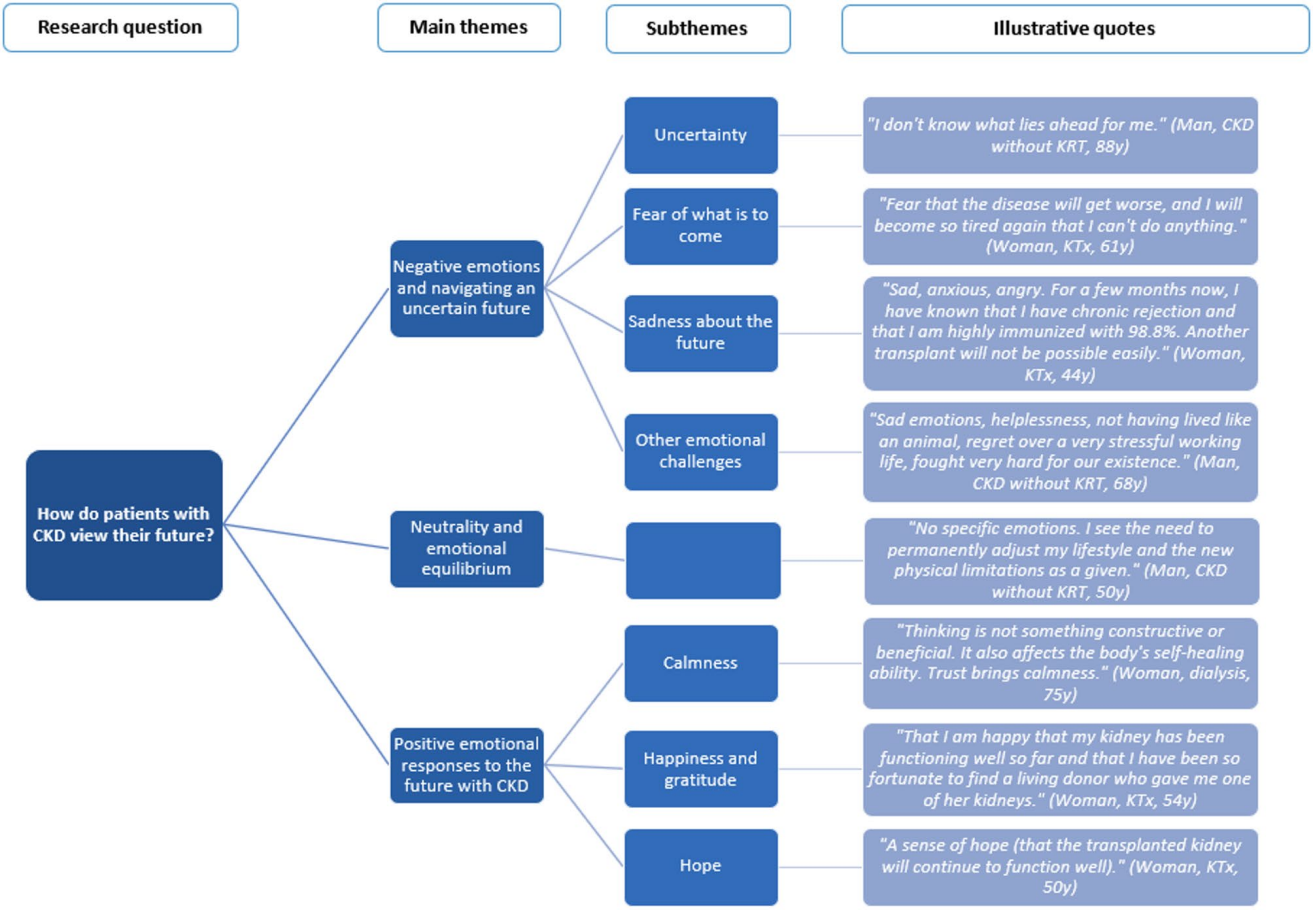
Visual representation of the findings for research question one

Three themes emerged and will be discussed below, with corresponding subthemes and illustrative quotes (see Fig. 1 for a visual representation and Table 2 for additional quotes).

**Table 2:** Illustrative quotes per (sub)theme for research question one: how do patients with chronic kidney disease view their future?

**Negative emotions and navigating an uncertain future**
** Uncertainty** - It could all be different again. (Man, KTx, 59y) - I wonder what is going to come my way. This mainly involves uncertainty and sadness. (Woman, CKD without KRT, 48y) - The uncertainty about the future, whether I will have to start dialysis and whether a donor kidney will be available for me at that time! (Man, CKD without KRT, 51y) - The uncertainty about how long my donor kidney will last? (Woman, KTx, 72y) - What if… my donor kidney stops, what would I want then… uncertain feeling. (Woman, KTx, 62y) ** Fear of what is to come** - I am very scared. (Woman, CKD without KRT, 79y) - Sometimes fear of how long the kidney will continue to function well. (Woman, KTx, 49y) - Fear of kidney rejection. (Woman, KTx, 69y) - What if my kidney fails, this is my second donor kidney. Quality of life will not improve. The fear that I will not grow old. (Woman, KTx, 51y) - Uncertainty, afraid of death. (Woman, CKD without KRT, 64y) - Fear of new medication routines and the structure/restrictions that come with it. (Woman, CKD without KRT, 31y) ** Sadness about the future** - Sadness about having to go back to dialysis (Man, KTx, 63y) - Sadness over things I can no longer do or am allowed to do (Woman, CKD without KRT, 31y) ** Other emotional challenges** - Furious. (Woman, CKD without KRT, 51y) - Resentment towards the general practitioner. (Man, KTx, 59y) - Vulnerability (Man, KTx, 67y) **Neutrality and emotional equilibrium** - None. I want to make the best of it. It doesn’t make me emotional. (Man, dialysis, 83y) - Nothing special. (Man, KTx, 75y) **Positive feelings regarding the future with CKD** ** Calmness** - I resign myself to it. (Woman, dialysis, 89y) - For now, I am calm and have reasonable trust in my body. (Woman, KTx, 57y) - Acceptance! (Man, dialysis, 77y) - No stress. (Man, KTx, 70y) - After finding a donor, I felt more peace of mind and greater confidence in the future. Now that I seem to be stable for a while, after medication and lifestyle changes, I am less focused on the future because the current stress is less present. (Woman, CKD without KRT, 60y) - Seize the day! (Woman, dialysis, 71y) - I want to make the best of it. (Man, dialysis, 83y) - I try to live day by day and enjoy every day. (Man, dialysis, unknown age) ** Happiness and gratitude** - Mainly that I am happy with the transplanted kidney I received. (Man, KTx, 70y) - Feelings of gratitude and joy that it has been going well for over thirty years. (Man, KTx, 38y) - Having received three transplants makes me very grateful and motivates me to handle this with great care. (Man, KTx, 73y) ** Hope** - It’s not a pleasant outlook, but acceptance and hope are also part of it. (Woman, dialysis, 71y) - Hopefully positive. ‘Glass half full.’ (Man, KTx, 68y) - I hope for a lot of luck to keep my new kidney functioning for a long time. (Man, KTx, 51y)

The first research question was answered using the following question from the survey: “What emotions do you experience when you think about your future with a CKD?”

### Negative emotions and navigating an uncertain future

Patients described a range of negative emotions, such as uncertainty, fear, and sadness. A predominant feeling was a profound sense of uncertainty regarding the future. Many patients reported a general uncertainty of what awaits:

*I don’t know what lies ahead for me. (Man, CKD without KRT, 88y)*

For other patients the uncertainty related to specific topics. For example, they wondered if and when they will need dialysis (again) or worried about the longevity of their donor kidney. Another common feeling was a general fear for their unknown future. Some were afraid of needing dialysis in the future, while others feared death or a short lifespan due to CKD. Another fear described was regarding CKD progression itself, leading to more severe health problems, new medication regimens and side effects, and worsening of symptoms:

*Fear that the disease will get worse*,* and I will become so tired again that I can’t do anything. (Woman*,* KTx*,* 61y)*

Notably, in some patients the worries motivated them to focus more on self-management:

*Then I really worry about what my life will look like. At the same time*,* this motivates me to take optimal care of myself and to influence what I can to slow down disease progression. (Woman*,* CKD without KRT*,* 60y)*

Sadness about future consequences of CKD was strongly present. For numerous patients it concerned an overall sadness without specific context. For others, sadness was related to their donor kidney potentially failing or the potential of having to undergo dialysis (again):

*Sad*,* anxious*,* angry. For a few months now*,* I have known that I have chronic rejection and that I am highly immunized with 98.8%. Another transplant will not be possible easily. (Woman*,* KTx*,* 44y)*

Other, less often described, negative emotions regarding their future included anger, helplessness, regret and vulnerability. For example, one participant stated feeling helpless and regretting having lived a life that was too stressful:

*Sad emotions*,* helplessness*,* not having lived life to the fullest*,* regret over a very stressful working life*,* fought very hard for our existence. (Man*,* CKD without KRT*,* 68y)*

### Neutrality and emotional equilibrium

Many patients reported not experiencing any particularly negative or positive emotions when thinking about their future. For some, this appeared to reflect a sense of emotional detachment toward their condition:

*No specific emotions. I see the need to permanently adjust my lifestyle and the new physical limitations as a given. (Man*,* CKD without KRT*,* 50y)*

For some patients, there appeared to be no reason for concern because their kidney function was currently stable:

*Given the stable course of my disease*,* currently I do not have major worries. (Man*,* CKD without KRT*,* 40y)*

### Positive emotional responses to the future with CKD

Patients shared an array of positive emotions such as calmness, hope and trust about their future with CKD.

A number of patients expressed a sense of calmness regarding their future. For example, patients described having accepted CKD and that they trust their body, bringing them a sense of calm:

*Thinking is not something constructive or beneficial. It also affects the body’s self-healing ability. Trust brings calmness. (Woman*,* dialysis*,* 75y)*

Some patients attempted to achieve this calmness by having a positive outlook on their future, enjoying their current life, living day by day, and making the best of it.

Patients also experienced feelings of happiness or hopefulness. For instance, finding a kidney donor, and donor kidney longevity evoked happiness and gratitude for several patients:

*That I am happy that my kidney has been functioning well so far and that I have been so fortunate to find a living donor who gave me one of her kidneys. (Woman*,* KTx*,* 54y)*

Hope was also experienced by patients: some held on to a general sense of hope regarding their future, while others hoped their donor kidney would function well for a long time:

*A sense of hope (that the transplanted kidney will continue to function well). (Woman*,* KTx*,* 50y)*

Finally, it should be noted that the themes reflect the range of emotions patients mentioned, and that some patients also described a mixture of positive and negative emotions.

## What are the prognostic needs of patients regarding their future with CKD? (RQ2)

**Fig. 2:**
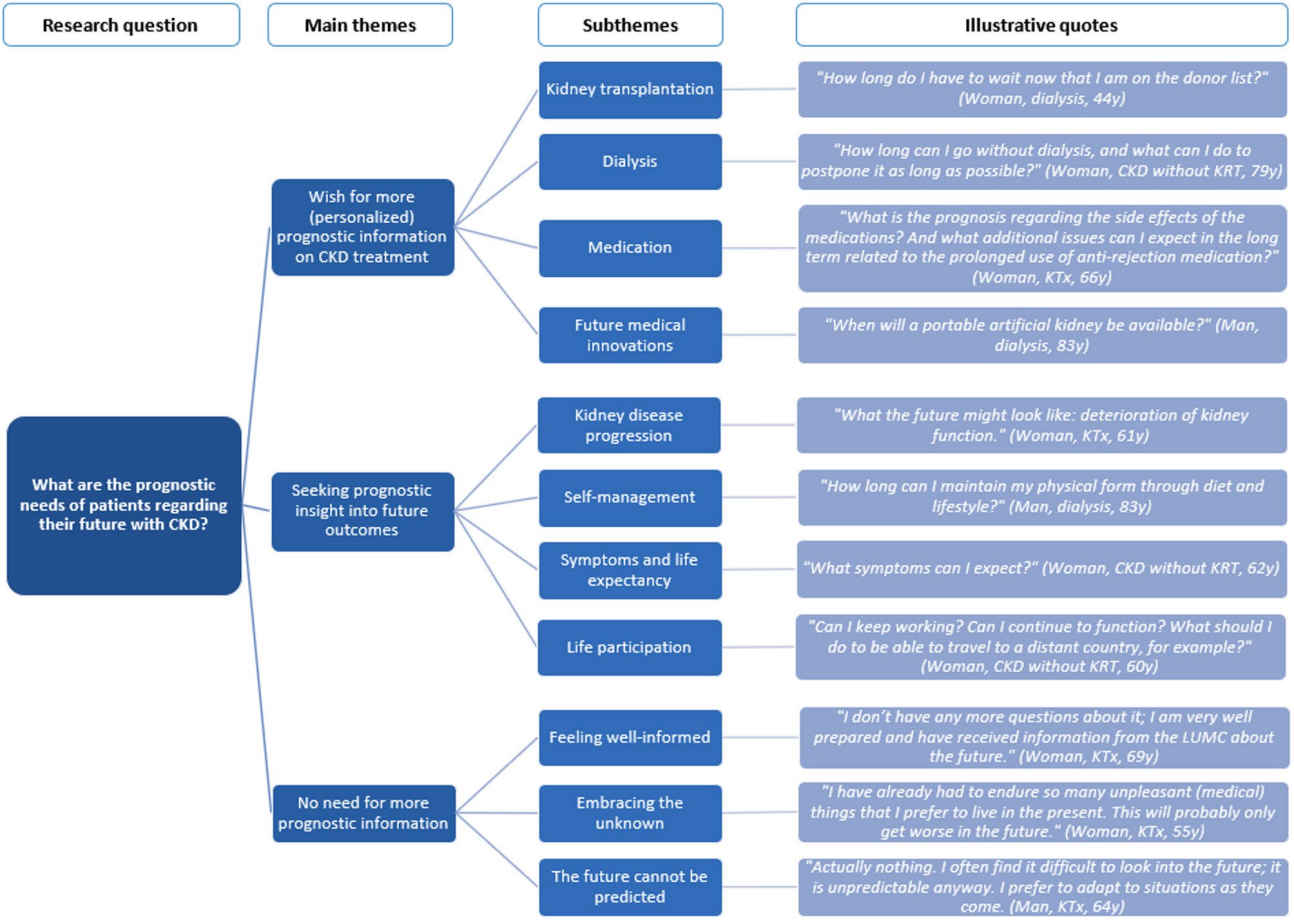
Visual representation of the findings for research question two

Many patients shared that they discuss ‘the future’ with healthcare professionals, covering a wide array of medical topics, such as treatment options, medication, physical symptom management, disease progression and prognosis. Only a few patients stated to discuss emotional and social consequences of CKD with healthcare professionals, like the impact on relationships, work participation, quality of life and mental health. The majority of patients shared a general wish for more personalized prognostic information on a variety of topics. Below, the three main themes are discussed, with corresponding subthemes and illustrative quotes (see Fig. 2; Table 3).

**Table 3:** Illustrative quotes per (sub)theme for research question two: what are the prognostic needs of patients regarding their future with chronic kidney disease?

**Wish for more (personalized) prognostic information on CKD treatment**
** Kidney transplantation** - Possibility of a potential new transplant. (Man, KTx, 67y) - How long will my donor kidney last? (Woman, KTx, 70y) - Is a transplant possible? What are the conditions? (Man, dialysis, 75y) - Risk of rejection. (Woman, KTx, 61y) - When will I receive a kidney? (Man, CKD without KRT, 52y) ** Dialysis** - Night dialysis; what are the possibilities. (Woman, CKD without KRT, 63y) - What are the options if I ever have to start dialysis again, and do I still want that? (Woman, KTx, 56y) - How long can I continue dialysis until a new donor becomes available? (Woman, dialysis, 64y) - Does home dialysis exist, and what does it entail? (Man, CKD without KRT,75y) ** Medication** - Optimizing medications to reduce side effects. (Woman, KTx, 61y) - Whether I can stop taking medications. (Woman, CKD without KRT, 63y) - Are there any expected changes in the medication policy? (Woman, KTx, 57y) - What can I expect regarding medications? (Woman, CKD without KRT, 62y) ** Future medical innovations** - Possible technological advancements in treatments. (Woman, dialysis, 71y) - Progress in the development of cures for kidney diseases. (Woman, KTx, 69y) - New, less burdensome medications. (Man, KTx, 70y) - Waiting for future developments in dialysis. (Man, dialysis, 77y) - Whether by the time I need to start dialysis, it might be a more pleasant experience, with fewer side effects, so that the rest of life can be more comfortable than how people currently feel after treatment. (Man, CKD without KRT, 51y) **Seeking prognostic insight into future outcomes** ** Kidney disease progression** - How long will my kidneys continue to function adequately? (Man, CKD without KRT, 75y) - Expected duration of good kidney function. (Man, KTx, 54y) - How long can I continue to live with my own kidneys? (Man, CKD without KRT, 66y) ** Self-management** - I would like to know if I can take more specific measures myself, for example in terms of diet, to maintain my health with the kidney as well as possible. (Woman, KTx, 65y) - What is the best I can do for the highest chance of a long life? (Man, KTx, 79y) - What can I do to keep my body as healthy as possible? (Woman, KTx, 54y) - Is there anything that can be done to slow down the procession? (Woman, CKD without KRT, 56y) ** Symptoms and life expectancy** - What symptoms can I expect? (Woman, CKD without KRT, 62y) - How long do I have left? (Man, KTx, 78y) - How old can I become? (Woman, CKD without KRT, 31y) - How long will I live this way? (Man, CKD without KRT, 67y) - How long can I continue to live relatively problem-free? (Woman, CKD without KRT, 48y) ** Life participation** - How long can I continue with my volunteer work? (Man, dialysis, 83y) - Financial security. (Man, KTx, 38y) **No need for more prognostic information** ** Feeling well-informed** - I already know a lot. (Man, KTx, 78y) - I have a general idea of what to expect. I read about this myself. (Woman, KTx, 40y) - I am fairly informed about what the future holds for me. (Man, CKD without KRT, 83y) ** Embracing the unknown** - I don’t know, I just experience it as it comes. (Man, dialysis, 86y) - I don’t want to know, because then I would plan my entire future around it. (Man, KTx, 70y) - Strangely, nothing. I manage well living day by day. (Man, KTx, 74y) ** The future cannot be predicted** - Nothing; no one can predict the future. I am stable. (Woman, KTx, 67y) - It has been going well for 31 years. The future is unpredictable anyway. (Woman, KTx, 66y) - Unfortunately, it cannot be predicted. (Woman, dialysis, 70y)

The second research question was answered using the following questions from the survey: “What would you like to know about your future with a CKD?”, and “If you think back to a year or two ago, what would you have wanted to know about your life with a CKD now?”

### Wish for more (personalized) prognostic information on CKD treatment

Patients mentioned being particularly interested in receiving personalized prognostic information about different treatment options, including dialysis, the risk of rejection and durability of their donor kidney, and the possibilities and timeline of receiving a kidney transplantation:

*How long do I have to wait now that I am on the donor list? (Woman*,* dialysis*,* 44y)*

Furthermore, thinking back to two years ago, they would have appreciated knowing more about their kidney graft. For instance, the durability of their donor kidney, whether they could have prevented chronic rejection, and the consequences of human leukocyte antigen (HLA) mismatches:

*The consequences of having many HLA mismatches on kidney survival. This has simply been discussed little or not at all with me. (Woman*,* KTx*,* 67y)*

Some patients were curious about the possibilities of different dialysis modalities, such as nightly dialysis or home dialysis, how long they can dialyze until a kidney donor comes available, or how to best prepare for dialysis. Others were interested in understanding the timeframe before dialysis and strategies to delay it:

*How long can I go without dialysis*,* and what can I do to postpone it as long as possible? (Woman*,* CKD without KRT*,* 79y)*

Patients were also seeking answers about their future medication regimen. They wondered whether they could stop taking certain immunosuppressive medications and what side effects to expect:

*What is the prognosis regarding the side effects of the medications? And what additional issues can I expect in the long term related to the prolonged use of anti-rejection medication? (Woman*,* KTx*,* 66y)*

Reflecting on the past years, many patients wished they had known more about the long-term consequences and side effects of immunosuppressive medication.

Patients also expressed an interest in the latest developments in CKD treatments, such as less burdensome dialysis techniques and medication, or innovative technologies like a portable artificial kidney:

*When will a portable artificial kidney be available? (Man, dialysis, 83y)*

### Seeking prognostic insight into future outcomes

Many patients were interested in gaining more insight into their prognosis, mainly how their kidney function will deteriorate in the future:

*What the future might look like: deterioration of kidney function. (Woman KTx, 61y)*

Patients also wanted to know more about strategies to slow down CKD progression. Patients asked several questions on how they could influence their prognosis by making lifestyle changes:

*How long can I maintain my physical form through diet and lifestyle? (Man, dialysis, 83y)*

Patients shared many questions about their overall health status in the future. Not only did they wonder what to expect in terms of their life expectancy with CKD, but also which symptoms they might experience in the future:

*What symptoms can I expect? (Woman, CKD without KRT, 62y)*

Additionally, patients were concerned about their societal and social roles, such as their ability to work/volunteer and travel:

*Can I keep working? Can I continue to function? What should I do to be able to travel to a distant country*,* for example? (Woman*,* CKD without KRT*,* 60y)*

### No need for more prognostic information

Some patients did not want to know more about their future. For example, some patients felt like they already had sufficient information about their future with CKD. Patients either read about CKD themselves or felt like they were adequately informed by their healthcare professionals:

*I do not have any more questions about it; I am very well prepared and have received information from the LUMC about the future. (Woman*,* KTx*,* 69y)*

Some patients preferred not to delve into the future and its uncertainties too much, and preferred to live in the present moment instead:

*I have already had to endure so many unpleasant (medical) things that I prefer to live in the present. This will probably only get worse in the future. (Woman*,* KTx*,* 55y)*

A few patients acknowledged the unpredictability of the future and emphasized they preferred to deal with any hurdle as it arises:

*Actually nothing. I often find it difficult to look into the future; it is unpredictable anyway. I prefer to adapt to situations as they come. (Man*,* KTx*,* 64y)*

## Discussion

In this qualitative study, we aimed to explore the following two research questions: (1) How do patients with CKD view their future? and (2) What are the prognostic needs of patients regarding their future with CKD? These questions address an important knowledge gap in understanding CKD patients’ future perspectives and prognostic needs. While more research now focuses on the development of prognostic models to predict the risk of various outcomes, it remains unclear what patients prefer regarding this prognostic information.

Regarding patients’ perspectives on the future, we found an array of emotional responses, from negative emotions like uncertainty, fear and sadness to positive ones like calmness, hope, and trust. Prognostic uncertainty in CKD poses considerable challenges, as supported by literature linking this uncertainty to anxiety, depressive symptoms and lower health-related quality of life [27, 28]. Interestingly, these negative emotions can serve a dual role: while burdensome, they may also be motivators for self-management, encouraging patients to find ways to delay disease progression [29]. Excessive fear or uncertainty, however, can become overwhelming and paralyzing [30–33]. Conversely, positive emotions, such as hope and trust, may serve as a psychological anchor, helping patients to deal with CKD-related uncertainties [15, 34]. Our findings underscore that acceptance plays an important role in maintaining a positive outlook, which aligns with previous research highlighting acceptance as a key coping strategy in CKD patients [35]. Moreover, psychological treatments that focus on acceptance, such as Acceptance and Commitment Therapy (ACT) have been shown to improve treatment adherence and reduce symptoms in dialysis patients [36, 37]. 

Additionally, some patients experience neither positive nor negative emotions, but rather a form of neutrality regarding their future. For some, this neutrality stems from stability of their disease, reducing concerns about the future. For others, it reflects emotional adjustment to and acceptance of the disease and associated challenges. Neutrality may serve as a coping mechanism, allowing patients to remain balanced and in control [35]. Finally, many patients experience a mixture of positive *and* negative emotions.

This wide variety and interplay of emotions reflects the psychological complexity of having CKD [16, 33]. This complexity can only be better understood through open conversations with patients, allowing healthcare professionals to gain awareness of the emotional challenges for patients with CKD. A holistic, patient-centred approach to nephrological care can encourage patients to engage more actively in shared decision-making and self-management, and can enhance mental and physical health outcomes [14, 38, 39]. 

Regarding prognostic needs, patients desired more personalized prognostic information on treatment strategies, including kidney transplantation, dialysis and medication. Although these topics are commonly discussed with healthcare professionals, it seems that these discussions sometimes lack detail and are not (sufficiently) tailored to the individual. Additionally, patients were interested in medical advancements (e.g., a portable artificial kidney or medication with less side effects), reflecting their hope for medical innovations and less burdensome treatment options [40, 41]. 

Patients also sought more information about prognostic outcomes such as CKD progression, life expectancy, and quality of life. A notable finding was the wish for more practical guidance on self-management strategies to prevent or slow down CKD progression. In order to actively engage in self-management, patients need knowledge on what they can do and how to do it [42]. The importance of self-management in CKD is increasingly recognized and literature shows that self-management interventions, consisting of, among other things, patient education, self-monitoring, motivational interviewing, and coaching, can indeed improve long-term outcomes [43, 44]. 

However, not all patients want to know more about what the future holds. Some patients feel sufficiently informed about their future, either by their healthcare providers or by the information they have sought out themselves. Others prefer to live in the present moment, as the future with CKD likely holds many unexpected challenges. Previous studies suggest that maintaining uncertainty may facilitate hope, especially when the alternative is negative certainty [45]. To maintain this uncertainty, patients may adopt coping strategies like avoidance, selective ignoring and neutralizing [46]. While these strategies might preserve short-term hope, they can also hinder patients from proactively engaging in self-management. At the same time, there is something to be said for living in the moment and learning to coexist with uncertainty, which can be an important aspect of psychological adaptation. Furthermore, while many patients expressed a desire for more personalized prognostic information, it is important to acknowledge that such information may also cause anxiety or can be misinterpreted. Patients may also fear bad news [47]. However, previous research in chronic illnesses suggest that carefully delivered prognostic information does not increase anxiety [48]. To enhance this process, it is essential that patients are supported in processing this information, for example through guidance from healthcare professionals. Importantly, patients should have a say not only in how much prognostic information they receive, but also in whether they wish to receive it at all, and considering these preferences is crucial in patient-centred care.

Thus, despite conversations about the future taking place between healthcare professionals and CKD patients, there remain unmet prognostic information needs. Our findings underscore the diversity and individuality of these prognostic needs, which is in line with our previous survey study showing that prognostic information needs differ based on CKD stage, gender and age [20]. In addition, these findings show the need for healthcare professionals to actively explore individual patients’ preferences regarding prognostic information, including how much detail they want, when they want it, how they receive such information and what emotions it may evoke. Tailoring these conversations can help patients feel more prepared for the future, improve emotional coping, and support shared decision-making. Clinicians should also be aware that some patients may choose not to receive future-oriented information, and this preference should be respected. Incorporating these insights into routine care can help patients engage in self-management, make informed choices, and feel more in control of their disease trajectory. Moreover, future research should focus on how healthcare professionals can facilitate these discussions about the future and how prognostic information can be tailored to the individual patient. Exploring the potential of digital tools for this purpose may also be valuable [49]. Furthermore, the relationship between patients’ emotional responses and coping strategies, and their prognostic information needs should be explored, providing more detailed guidance for personalized care. In addition, qualitative studies using open-ended discussion (e.g. interviews or focus groups) could provide more detailed insights into the personal narratives, emotions, and contextual factors that shape patients’ views on their future. For example, such studies could help to better understand why some patients report emotional neutrality when thinking about their future with CKD.

To our knowledge, this study is among the first to delve further into the future perspectives and prognostic needs of patients with CKD. A strength of this study is that we included patients from all stages of CKD to offer a broad perspective on this topic, and the large sample for a qualitative study. However, this study also has some limitations. First, only patients from Dutch patient associations and hospitals were included, and the survey was only available in Dutch. Therefore, transferability of results to other patient groups (e.g., who do not speak the Dutch language) and other countries, is limited. Additionally, kidney transplant recipients were overrepresented in our sample (56%), which may impact transferability of our results. Second, researchers’ preconceptions, experiences and expectations may have coloured the interpretation of results. To address this, a group of researchers with a diverse background were involved throughout the course of this study. Third, we used open-ended survey questions, which do not allow for the same depth of exploration as is possible using open-ended discussions (e.g. interviews or focus groups), as it was not possible to ask follow-up questions. As a result, some nuances and complexities of patients’ experiences may have remained unexplored. However, this method allowed us to include a large and diverse group of CKD patients across all disease stages, and we were able to capture less common experiences and emotions that might not have surfaced in a smaller interview study. Furthermore, some patients may feel more comfortable writing about sensitive topics than discussing them face-to-face. Fourth, some questions required patients to reflect on what they would have wanted to know earlier in their disease trajectory. Their current experiences, however, may have influenced how they remember or reinterpret their earlier information needs and perspectives. This may have affected the way current findings represent patients’ earlier perspectives. Fifth, the response rate of approximately 20% may have introduced non-response bias, as responders might differ in important ways from those who chose not to respond, limiting transferability. For example, patients who are more engaged in clinical research or who are more comfortable thinking about the future may have been more likely to participate. Finally, coding of the data was primarily conducted by a single researcher. However, this was done in close consultation with a second researcher during the initial phases to discuss interpretations. Furthermore, to enhance the trustworthiness of the results, regular consensus meetings were held in which findings were discussed within a multidisciplinary team of clinicians, psychologists and researchers. Team members were experienced in working with CKD patients and were familiar with relevant literature. This collaborative approach aligns with the principles of investigator triangulation, supporting the credibility of the results.

## Conclusion

To conclude, patients with CKD experience a wide variety of emotions regarding their future, and despite conversations about the future taking place in nephrological practice, there remain unmet questions regarding the future. While many patients expressed a wish for more personalized prognostic information on several topics, others preferred to avoid gaining more knowledge on their future. These results underline the importance of adopting a personalized approach to prognostic information provision for patients with CKD, acknowledging and taking into account the diverse emotional responses and individual preferences of patients.

## Electronic supplementary material

Below is the link to the electronic supplementary material.

Supplementary Material 1

Supplementary Material 2

## Data Availability

An Excel file with all data accompanied by a data dictionary can be shared on reasonable request to the corresponding author.
