# Supplementary material for: Living with chronic kidney disease: future perspectives and prognostic needs of patients - a qualitative study
Source: BMC Nephrol. 2025 Jul 1;26:301. doi: 10.1186/s12882-025-04243-8 (PMC12211900; doi:10.1186/s12882-025-04243-8)
Supplement: Supplementary file 1 — Supplementary Material 1 [file 12882_2025_4243_MOESM1_ESM.docx]

Research shows that people with chronic kidney disease often feel uncertain about their future. Dealing with a kidney condition brings many challenges. This can raise various questions. We kindly invite you to participate in our survey. The aim of our survey is to assess whether people with kidney disease need more information about their future with the illness. Additionally, we want to understand the specific questions people with chronic kidney disease have about their future. By doing so, we hope to enhance the education provided in the doctor's office, enabling doctors to deliver better personalized care for people with chronic kidney disease.

**Survey**

**“What do people with chronic kidney disease want to know about their future?*”***

Filling out the survey will take approximately 15 minutes. You can complete the survey online using the link provided below or via the QR code, or on paper (see pages following this one). Thank you in advance for your participation! Feel free to share the survey with other people who have a chronic kidney disease as well.

**URL to the survey:**

**What happens to your data?**
Your responses to the survey will remain completely anonymous. This means that nobody can see who has filled out the survey. Your own doctor will not have access to your answers either. We will treat the results confidentially and only use them for research purposes.

**Would you like to know anything else?**
Do you have any questions about the survey after reading this information? If so, you can contact the principal investigator, Jet Milders, at j.milders@lumc.nl.

**What is your gender?**

***General questions***

Male

Female

Other

**What is your age in years?**

**What is the highest level of education you have completed?**

No education

Primary education (elementary school)

Lower secondary education

Upper secondary education

Short-cycle tertiary education

University of applied sciences

Academic university

Other, please specify...

**What is your living situation?**

*(You can check multiple boxes if applicable)*

I live alone

I live with my partner

I have children living at home

I live with one of my children

I live with my parent(s)

I live in sheltered housing

I live in a care facility

Other, please specify...

**How long have you known that you have a kidney disease?**

Less than 1 year

1-4 years

5-10 years

More than 10 years

**Do you know the underlying cause of your kidney disease?**

I do not know

No cause has been found

Diabetes mellitus

Vascular disease (including high blood pressure)

Glomerulonephritis (inflammation of the kidney filters)

Pyelonephritis, kidney damage due to medication or kidney stones

Polycystic kidney disease

Systemic disease (e.g., lupus, hemolytic uremic syndrome)

Cancer

Other, please specify...

**Do you know your approximate kidney function?**

No, I do not know what my kidney function is

60 or higher

45-59

30-44

15-29

Less than 15

**What treatment are you undergoing and/or have you undergone?**

*(You can check multiple boxes if applicable)*

No treatment

Medication, diet, and/or lifestyle advice

I have undergone a kidney transplantation

I am on the waiting list or in the pre-transplant phase for a transplantation

I have undergone dialysis in the past

I am currently undergoing dialysis

I am receiving conservative management (instead of dialysis)

Other, please specify...

***Please only answer this question if you chose "I am currently undergoing dialysis" in the previous question:***

**What type of dialysis are you receiving?**

Hemodialysis in a center/hospital

Home hemodialysis

Peritoneal dialysis

**How many (online) appointments have you had with your nephrologist for your kidney disease in the past year?**

I have not had any appointments with my nephrologist in the past year

1-4 times in the past year

5-10 times in the past year

Every month

Every week

**Do you ever think about your future with a kidney disease?**

***The following questions are about your future with a kidney disease.***

Yes, I think about this often

Yes, I think about this every now and then

No, I never think about this

***Please only answer this question if you chose "Yes, I think about this often" or "Yes, I think about this occasionally" in the previous question:***

**What feelings do you experience when you think about your future with a kidney disease?**

**Have you ever discussed your future with kidney disease with your treating physician?**

Yes
 No, but I want to
 No, I do not want to
 Other, please specify...

***Please only answer this question if you chose "Yes" in the previous question:*Could you elaborate on what the conversation was about?**

**What would you like to know about your future with a kidney disease?**

**If your doctor had more information about your future, would you want to know?**
*(You can check multiple boxes if applicable)*

***For one person, a kidney disease may progress differently than for another. While it is not possible to predict the future with certainty, it is often possible to make an individual assessment. For example, a doctor may estimate the likelihood of someone needing to start dialysis within a year.***

I always want to know, even if it's bad news

I only want to know when it's good news

I want to know if there's something we can do to prevent it

I want to know to help me make choices about my treatment

I am not sure if I would want to know

I prefer not to know

Other, please specify...

***If you chose "I am not sure if I would want to know" in the previous question:*Why are you unsure if you would want to know?**

***If you chose "I prefer not to know" in the previous question:*Why do you prefer not to know?**

***Symptoms***

***You will now be shown a number of categories. Suppose you could get more information about these topics in the future. How much would you like to know more about these categories regarding your expected future (related to your kidney disease)?***

***This is not about which topics you generally find important, but specifically what aspects of your future you would like to know more about.***

**How much would you like to know more about symptoms in the future?***(Please indicate your answer by placing a cross on the line below)*

**Very much
(100)**

**Not
at all
(0)**

**(0**

**Which specific topics related to symptoms would you like to know more about in the future?***(You can check multiple boxes if applicable)*

None
 Pain
 Neuropathy
 Itching
 Fatigue
 Restless legs
 Tingling sensations
 Dizziness
 Nausea
 Muscle cramps

Sleeping problems
 Coughing
 Chest pain
 Dry skin
 Gastrointestinal issues
 Sexual problems
 Weight loss/gain
 Medication side effects
 Other, please specify...

**How much would you like to know more about disease progression and comorbidities in the future?***(Please indicate your answer by placing a cross on the line below)*

***Disease progression and comorbidities***


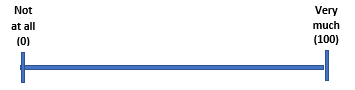


**Which specific topics related to disease progression and comorbidities would you like to know more about in the future?***(You can check multiple boxes if applicable)*

None
 Cardiovascular disease
 Infection
 Cancer
 Diabetes mellitus
 COVID-19
 Kidney disease progression

Life expectancy with the kidney disease
 Hospitalization
 Heritability of the disease / passing it on to children
 Other, please specify...

***Dialysis***

**How much would you like to know more about dialysis in the future?***(Please indicate your answer by placing a cross on the line below)*


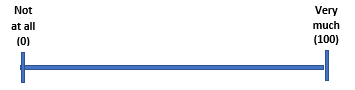


**Which specific topics related to dialysis would you like to know more about in the future?***(You can check multiple boxes if applicable)*

None
 Will I ever need to undergo dialysis again?
 When do I need to start dialysis?
 How long do I need to undergo dialysis?
Which dialysis method (hemodialysis or peritoneal dialysis) is most suitable for me
 Is nocturnal dialysis an option for me?
 How effective is dialysis?
 How long will I survive on dialysis?
 Which vascular access for dialysis is most suitable for me?
 Will I experience peritonitis?
 Other, please specify...

***Kidney transplantation***

**How much would you like to know more about kidney transplantation in the future?***(Please indicate your answer by placing a cross on the line below)*


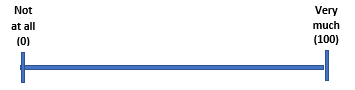


**Which specific topics related to kidney transplantation would you like to know more about in the future?***(You can check multiple boxes if applicable)*

None
 Will I receive a kidney transplant?
 When will I receive a kidney transplant?
 What type of donor kidney (living or deceased donor) will I receive?
 Will I experience complications from the surgery?
 How long will I survive after a kidney transplantation?
 What is the function of my donor kidney?
 Will my body reject the donor kidney?
 Will my donor kidney become infected?
 Will I experience side effects of immunosuppressive medications?
 Other, please specify...

***Conservative management***

*Conservative management means choosing not to start dialysis and not proceeding with a kidney transplantation. However, symptoms are treated with medication and lifestyle advice.*

**How much would you like to know more about conservative management in the future?***(Please indicate your answer by placing a cross on the line below)*


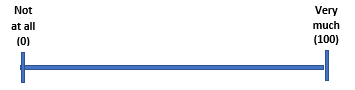


**Which specific topics related to conservative management would you like to know more about in the future?***(You can check multiple boxes if applicable)*

None
 Is conservative management an option for me?
 How long will I survive with conservative management?
 What symptoms will I experience if I choose conservative management?
 Other, please specify...

***Laboratory values and measurements***

**How much would you like to know more about laboratory values and measurements in the future?***(Please indicate your answer by placing a cross on the line below)*


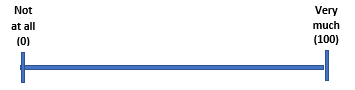


**Which specific topics related to laboratory values and measurements would you like to know more about in the future?***(You can check multiple boxes if applicable)*

None
 Kidney function
 Protein in the urine
 Blood pressure
 Potassium levels
 Phosphate levels

Calcium levels
 Hemoglobin levels (anemia)
 Other, please specify...

***Physical well-being***

**How much would you like to know more about your physical well-being in the future?***(Please indicate your answer by placing a cross on the line below)*


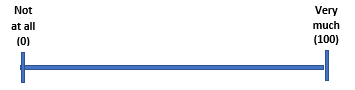


**Which specific topics related to physical well-being would you like to know more about in the future?***(You can check multiple boxes if applicable)*

None
 Energy levels
 Activities of daily living (bathing, dressing, etc.)
 Moderate physical activity (walking stairs, household chores, walking, cycling, etc.)
 Intensive physical activity (sports)
 Diet
 Other, please specify...

***Mental well-being***

**How much would you like to know more about your mental well-being in the future?***(Please indicate your answer by placing a cross on the line below)*


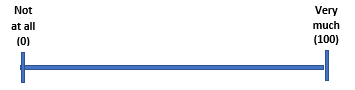


**Which specific topics related to mental well-being would you like to know more about in the future?***(You can check multiple boxes if applicable)*

None
 Quality of life (related to the kidney disease)
 Mood
 Stress or anxiety
 Nervousness
 Depression
 Concentration issues
 Memory issues
 Other, please specify...

***Social participation***

**How much would you like to know more about your social participation in the future?***(Please indicate your answer by placing a cross on the line below)*


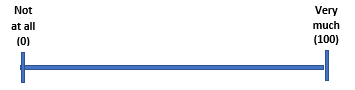


**Which specific topics related to social participation would you like to know more about in the future?***(You can check multiple boxes if applicable)*

None
 Will I still be able to work?
 Will I still be able to volunteer?
 Will my income decrease?
 Will I still be able to go on vacation?
 What will be the impact of the disease on my social life?
 What will be the impact of the disease on my family?
 Will I be able to have children?
 Will I still be able to live independently?
 Will I still be able to pursue my hobbies?
 Other, please specify...

**If you think back to a year or two ago, what would you have wanted to know about your life with a kidney disease now?**

**If there are any other matters left unaddressed, or if you have any further questions or would like to provide additional feedback, please feel free to do so.**

**This is the end of the survey. Once again, thank you very much for your time and attention!**
